# Bone Scintigraphy After a Negative Radiological Skeletal Survey Improves the Detection Rate of Inflicted Skeletal Injury in Children

**DOI:** 10.3389/fped.2020.00498

**Published:** 2020-09-25

**Authors:** Flora Blangis, Cyrielle Poullaouec, Elise Launay, Nathalie Vabres, Flavie Sadones, Thomas Eugène, Jérémie F. Cohen, Martin Chalumeau, Christèle Gras-Le Guen

**Affiliations:** ^1^Obstetrical, Perinatal and Pediatric Epidemiology Research Team, Epidemiology and Statistics Research Center, Université de Paris, INSERM, Paris, France; ^2^INSERM CIC 1413, Nantes University Hospital, Nantes, France; ^3^Department of General Pediatrics and Pediatric Infectious Diseases, AP-HP, Necker-Enfants Malades Hospital, Université de Paris, Paris, France; ^4^Department of Pediatric Emergency Care, Nantes University Hospital, Nantes, France; ^5^Unité d'Accueil des Enfants en Danger, Nantes University Hospital, Nantes, France; ^6^Department of Radiology, Nantes University Hospital, Nantes, France; ^7^Department of Nuclear Medicine, Nantes University Hospital, Nantes, France

**Keywords:** child abuse, diagnosis, skeletal injury, radiological skeletal survey, bone scintigraphy, add-on test

## Abstract

**Background:** Timely diagnosis of child physical abuse is of paramount importance. The added value of bone scintigraphy (BS) after a negative radiological skeletal survey (RSS) in children with suspected physical abuse has never been evaluated.

**Objective:** The objective of this study was to assess the extent to which BS could improve the detection rate of skeletal injury in children with suspected physical abuse with an initial negative RSS.

**Methods:** We used discharge codes to retrospectively identify children evaluated for suspected physical abuse in a university hospital (Nantes, France). We included all consecutive children younger than 3 years old who underwent both RSS and BS, with an interval of ≤96 h between tests, from 2013 to 2019. BS and RSS results were interpreted independently during the study period. We specifically analyzed BS results for children with a negative RSS to assess the value of BS as an add-on test.

**Results:** Among the 268 children ≤3 years old with suspected physical abuse who underwent RSS, 140 (52%) also underwent BS within 96 h and were included in the analysis. The median age was 6 months old (interquartile range: 3–8). The detection rate of ≥1 skeletal injury with RSS alone was 49% (*n* = 69/140, 95% CI: 41–58%) vs. 58% (*n* = 81/140, 50–66%) with RSS followed by add-on BS, for an absolute increase in the detection rate of 9% points (95% CI: 4–14%). The number of children with a negative RSS who would need to undergo BS to detect one additional child with ≥1 skeletal injury was 6 (95% CI: 4–11).

**Conclusion:** In young children with suspected physical abuse with a negative RSS, add-on BS would allow for a clinically significant improvement in the detection rate of skeletal injuries for a limited number of BS procedures required. Prospective multicenter studies are needed to confirm these findings.

## Evidence Before This Study

Uncertainty about the extent of the added value of bone scintigraphy (BS) after a negative radiological skeletal survey (RSS) impedes harmonized guidelines on the role of BS in the diagnostic pathway of young children with suspected physical abuse.

## Added Value of This Study

Performing BS in young children with suspected physical abuse after a negative RSS would allow for a clinically significant improvement in the detection rate of skeletal injuries for a limited number of BS procedures required.

## Introduction

Physical abuse is estimated to occur in 4–16% of the population younger than 18 years old in high-income countries (1). Timely diagnosis of child physical abuse is of paramount importance because failure to identify an inflicted injury and to intervene appropriately may put the child at risk for further abuse and precludes treatment of injuries and rehabilitation (2–5). After cutaneous injuries, inflicted skeletal injuries are the second most common finding in children with suspected physical abuse (3, 6–10) and are an indicator of severe assault (8–13).

Radiological skeletal survey (RSS) is the currently recommended test for the diagnosis of inflicted skeletal injuries in all children under 2 years old with suspected physical abuse (6, 13–16). However, RSS cannot be considered a reference standard because skeletal injuries of the ribs, hands, and feet, which are reliable indicators of child physical abuse (8–13, 17), may be inconspicuous or invisible on initial RSS (18–21). Thus, follow-up RSS after 2 weeks may help detect skeletal injuries missed on the initial RSS (14, 18, 19, 21) and are recommended (6, 13–16). Several reports have underscored the lack of adherence to follow-up RSS, mainly because of concerns about delaying the diagnosis (22–25). Any alternative approach that would enhance the detection rate of inflicted skeletal injuries without causing a 2-weeks delay would be clinically highly relevant.

Preliminary reports have suggested that bone scintigraphy (BS) with technetium-99m diphosphonate can increase the detection rate with RSS (26–31). Indeed, BS presents a functional evaluation of total skeletal metabolism, which allows for detecting a skeletal injury within hours of its occurrence, and has shown high diagnostic performance in the assessment of various bone disorders (32). BS has poor sensitivity for “classical metaphyseal injuries” and other frequent skeletal injuries considered typical of child physical abuse (29, 31) because of physiological hypermetabolism in these regions, thereby capturing the radiotracer. Therefore, BS cannot replace RSS. Moreover, BS implies exposure to more radiation than does RSS (2.3 vs. 0.1–0.5 mSv on average) (33), is available only in hospitals with nuclear medicine departments, and adds costs. Positioning BS as an add-on test (34) after a negative RSS would allow for using the reclassification potential of BS while limiting the number of BS procedures performed. The benefit of this strategy has never been evaluated.

The objective of this study was to assess the extent to which BS could improve the detection rate of skeletal injury in children with suspected physical abuse with an initial negative RSS.

## Methods

### Study Design

We conducted a retrospective study in a tertiary university pediatric hospital. The protocol was approved by the ethics committee of Nantes University Hospital, allowing for this observational study with information for parents available on billboards placed in all public places of the hospital. We used the Strengthening the Reporting of Observational Studies in Epidemiology (STROBE) guidelines (35) to report this study (Appendix 1).

### Participants

We used the following ICD-10 discharge codes to retrospectively identify, among all children evaluated by RSS, those potentially evaluated for a suspicion of physical abuse: abuse syndromes (codes beginning with T74), intracranial traumatic injuries (codes beginning with S06), intra-abdominal traumatic injuries (codes beginning with S36), and skeletal injuries (codes beginning with S02, S12, S22, S32, S42, S52, S62, S72, S82, S92, M84). We included the medical files of all consecutive children younger than 3 years old who underwent both RSS and BS, from January 2013 to July 2019, a period during which the local protocol encouraged clinicians to perform both tests in children with suspected physical abuse. We included children for whom tests were performed with an interval of ≤96 h to limit bias related to time.

### Imaging Tests

During the study period, the local protocol for RSS agreed with international guidelines (6, 15, 16, 36) with performance of frontal and profile skull views, frontal chest view, profile whole spine views, frontal unprepared abdominal view, frontal upper-limb (arms, forearms, hands) views, frontal lower-limb (thighs, legs, feet) views, and frontal pelvis views. A profile incidence of the chest was added with suspected rib injuries. The local protocol for BS also agreed with international guidelines (32). The acquisition was carried out in the “whole body” twice after the injection of the radiotracer (99m-Tc diphosphonate): first, the tissue acquisition at 5 min after the injection and, second, the bone acquisition at 3–4 h after the injection. The doses of the radiotracer were adapted to the child's weight, with a baseline activity of 35.0 MBq (32). BS was performed *in situ* without sedation.

BS and RSS results were interpreted independently during the study period. All RSS results were analyzed by a senior pediatric radiologist, and all BS results were analyzed by a senior physician in nuclear medicine, with strong expertise in RSS and BS, respectively. In our study, we used the results of the routine interpretation of both tests. Positivity of the RSS or BS was defined as the detection of one or more skeletal injuries (i.e., fracture, periosteal apposition, displacement) in a child, including any index skeletal injury(ies), that is, the skeletal injury(ies) detected before RSS, which were at the origin of the suspected physical abuse and its diagnostic workup. A negative RSS or BS was defined as no skeletal injury detected. When the BS result was considered doubtful, new radiographs were obtained 7–10 days later on the doubtful region. No muscle injury was reported.

### Data Extraction and Analyses

Two of the authors (FB and CP) reviewed the medical files and extracted the children's characteristics (age at the time of the imaging investigation, sex, reason for suspecting physical abuse), the imaging protocol followed, and the number of skeletal injuries detected by RSS and BS and their location. We calculated the detection rates of children with ≥1 skeletal injury with RSS alone and with add-on BS (i.e., after a negative RSS), the absolute increase in the detection rate of children with ≥1 skeletal injury with the add-on strategy, and the number needed to test (NNT) (i.e., the number of children with a negative RSS who would need to undergo a BS to detect one additional child with ≥1 skeletal injury; formula in Appendix 2). We also evaluated the number of additional skeletal injuries detected by BS, their location, and whether they led to an orthopedic or surgical treatment. Finally, we described the number of additional skeletal injuries detected by BS for children after a positive RSS.

We conducted two sensitivity analyses. In the first, we included only children with an interval between tests of ≤24 h. In the second, we included children with an interval between tests of ≤96 h, but outcomes were recalculated considering the RSS as negative if it detected no skeletal injury or only an index skeletal injury (i.e., those already known before RSS and at the origin of the suspected physical abuse and its diagnostic workup). We used R v3.5.2 for the analyses (R foundation for Statistical Computing, Vienna, Austria).

## Results

### Population

**Figure 1:**
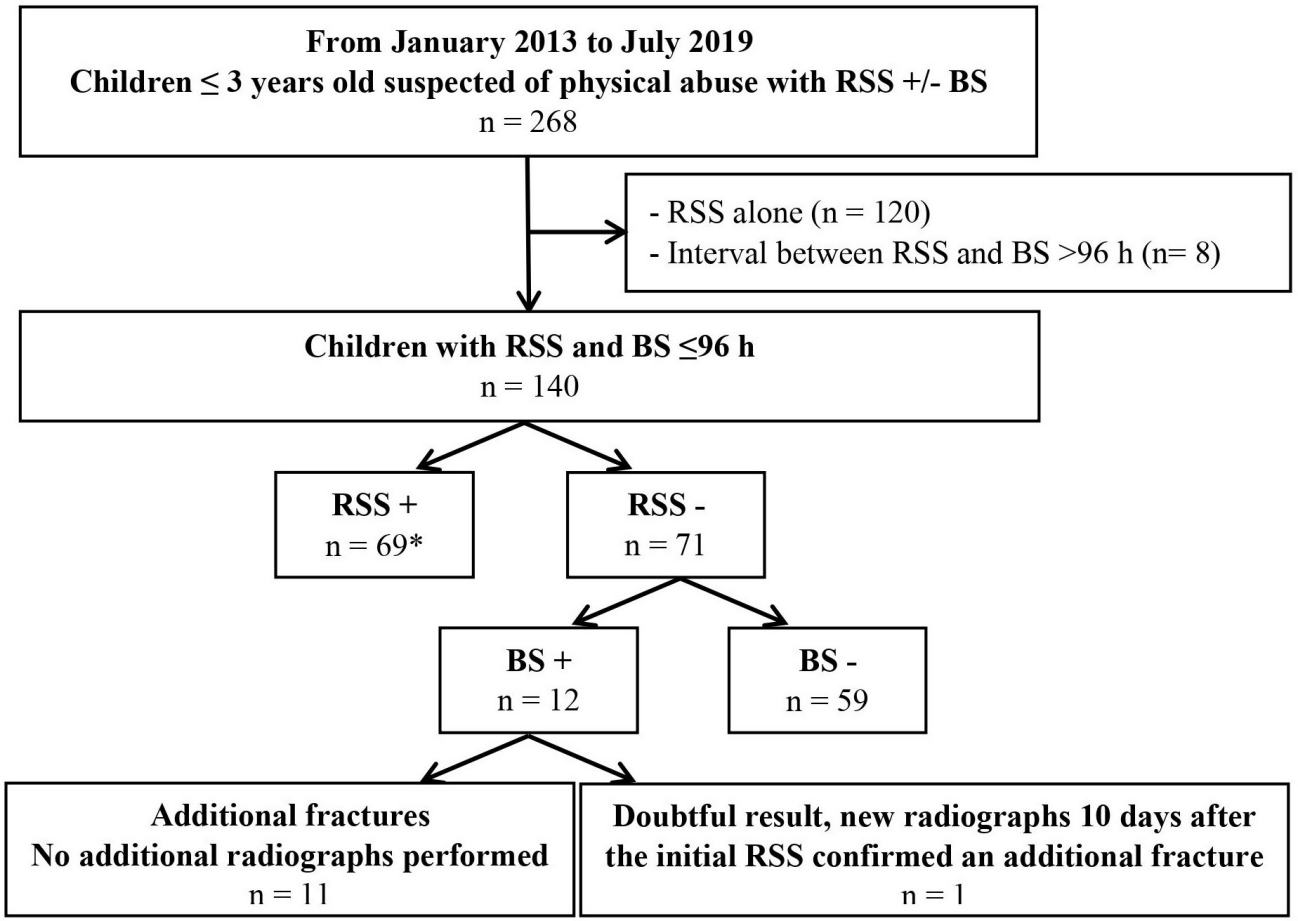
Flow chart of inclusion of children and results of radiological skeletal survey (RSS) and add-on bone scintigraphy (BS). *Among the 69 children with positive RSS, BS detected 48 supplementary skeletal injury in 34 children (34/69, 49%).

During the study period, among the 268 children ≤3 years old with suspected physical abuse who underwent RSS, 140 (52%) also underwent BS within 96 h (Figure 1) and were included in the analysis. The median age was 6 months (interquartile range: 3–8), and 54% (*n* = 75) were boys. The main reasons for suspecting physical abuse were index skeletal injuries (44%, *n* = 61), bruises (35%, *n* = 49), and/or intracranial injuries (24%, *n* = 34) (Appendix 3). Children who underwent BS differed significantly from those who did not only in the reason for suspecting physical abuse: they had significantly more intracranial injuries than those who did not undergo BS (24%, *n* = 34, vs. 10%, *n* = 12, *p* = 0.01) (Appendix 3).

### Imaging Tests

RSS and BS were performed within 72 h for 97% (*n* = 136/140) of the children and within 24 h for 76% (*n* = 106/140) of the children. The detection rate of ≥1 skeletal injury with RSS alone was 49% (*n* = 69/140; 95% confidence interval [CI] 41–58%) and revealed a total of 132 skeletal injuries (range: 1–12) (Figure 1 and Table 1). The detection rate of children with ≥1 skeletal injury with the add-on BS strategy was 58% (*n* = 81/140; 50–66%), revealing skeletal injuries in 12 children among those with negative RSS and 23 new skeletal injuries (range: 1–4 skeletal injuries). The absolute increase in the detection rate with add-on BS was 9% points (95% CI: 4–14%) and was statistically significant (McNemar test, *p* = 0.001). The NNT to detect one additional child with ≥1 skeletal injury was 6 (95% CI: 4–11). Among the 23 skeletal injuries not detected by RSS, the most frequent locations were the foot (17%, *n* = 4) and tibia (17%, *n* = 4) (Table 1). New skeletal injuries were revealed by BS after a negative RSS in 12 children. In 11 of these 12 children, the diagnosis of skeletal injury was definitively made on BS alone (Figure 1). For one child, BS was inconclusive, which led to obtaining new radiographs of the doubtful region 10 days after the initial RSS. These new radiographs confirmed one skeletal injury. Among these 12 children, the additional injuries identified did not lead to any orthopedic or surgical treatment.

**Table 1:** Location of the skeletal injuries detected by radiological skeletal survey and add-on bone scintigraphy in the 140 included children.

**Skeletal injury location**	**Detection with radiological skeletal survey**		**Additional detection with add-on bone scintigraphy**	
	**Number of skeletal injuries No. (%)**	**Number of children with ≥1 skeletal injuries No. (%)**	**Number of skeletal injuries No. (%)**	**Number of children with ≥1 skeletal injuries No. (%)**
Skull	18 (13.6)	14 (20.3)	1 (4.3)	1 (8.3)
Clavicle	5 (3.8)	5 (7.2)	0	0
Humerus	7 (5.3)	7 (10.1)	0	0
Radius	8 (6.1)	7 (10.1)	3 (13.0)	2 (16.7)
Ulna	5 (3.8)	4 (5.8)	2 (8.7)	2 (16.7)
Hand	2 (1.5)	1 (1.4)	0	0
Thoracic cage	29 (22.0)	7 (10.1)	2 (8.7)	2 (16.7)
Spine	0	0	2 (8.7)	1 (8.3)
Pelvis	0	0	2 (8.7)	2 (16.7)
Femur	28 (21.2)	22 (31.9)	2 (8.7)	2 (16.7)
Tibia	25 (18.9)	21 (30.4)	4 (17.4)	4 (33.3)
Fibula	5 (3.8)	4 (5.8)	0	0
Foot	0	0	4 (17.4)	4 (33.3)
Total	132	69^*^	23	12^†^

* *69 children had a positive radiological skeletal survey. Results do not total 100% because some children had skeletal injuries in different locations*.

† *12 children had a positive bone scintigraphy. Results do not total 100% because some children had skeletal injuries in different locations*.

### Sensitivity Analyses

On sensitivity analysis including only children with an interval between tests of ≤24 h (76%, *n* = 106), the RSS detection rate of children with ≥1 skeletal injury was 52% (*n* = 55/106; 42–62%), with an absolute increase in the detection rate of 8% points (95% CI: 4–16%) with add-on BS and an NNT of 6 (95% CI: 3–12) (Appendix 4).

For 44% (*n* = 61) of the children, physical abuse was initially suspected on the basis of ≥1 index skeletal injury(ies). By defining a positive RSS when it detected ≥1 skeletal injury different from the index skeletal injury(ies), the RSS detection rate of children with ≥1 skeletal injury(ies) was 22% (*n* = 31/140; 16–30%), with an absolute increase in the detection rate of 14% points (95% CI: 8–20%) with add-on BS and an NNT of 6 (95% CI: 4–9) (Appendix 4).

## Discussion

### Key Results

The stakes of identifying skeletal injuries in a child with suspected physical abuse are critical, with major impact on the early decision for child protection and subsequent consequences for the early and long-term health of the child. Thus, our objective was to evaluate the added value of BS after a negative RSS to detect skeletal injuries in children with suspected physical abuse. The absolute increase in the detection rate was 9% points and was statistically significant. This increase in the detection rate was obtained by performing BS in only half of the children with suspected physical abuse. The add-on strategy allowed for limiting the number of BS procedures performed, with an NNT of 6. The extent of the increase in the detection rate was similar to or higher on both sensitivity analyses performed with a more stringent maximal interval between the two tests (≤24 h) or by defining a positive RSS when it detected ≥1 skeletal injury different from the index skeletal injury(ies). Regarding the benefit–inconvenience balance, this low number of necessary BS procedures seems compatible with the burden of this procedure, which involves added radiation exposure (2.3 mSv on average) and requires intravenous access and sometimes a transfer to another hospital with a nuclear medicine department.

### Limitations

Our study had limitations. First, it was retrospective and performed in a single tertiary hospital with a referral center for abused children. This design could have led to a biased selection of children more seriously affected. Only 55% of the eligible children underwent both RSS and BS in the study period, which suggests a higher medical worry for those children and was confirmed by the higher rate of intracranial injuries than in children with RSS alone (Appendix 3). Subsequently, the NNT calculated in the present population could be biased and would be higher with a less selected population. However, the general characteristics of the study population were close to those reported in the literature (37–41) in terms of age, sex ratio, and frequency of skeletal injuries.

The interpretation of the RSS may be difficult, and a new interpretation by one or more independent experts may have led to the detection of more skeletal injuries. However, the choice not to reanalyze the RSS allowed for evaluating the impact of BS in “real life,” not in an experimental situation.

We could not estimate the exact added value of BS to reclassify child physical abuse. Indeed, the final diagnosis of child physical abuse relies on a combination of social and clinical evaluations and medical tests (42), so the detection of additional skeletal injuries by BS may have limited impact when cutaneous, neurological, or ophthalmologic findings suggesting physical abuse have already been detected. However, the additional skeletal injuries detected by BS may confirm the diagnosis of abuse in doubtful cases, and their location brings additional information to adapt treatment (43) and evaluate the potential mechanisms involved in the inflicted injury (20, 42).

Finally, we focused on an add-on strategy among children with a negative RSS and did not emphasize the 69 children with a positive RSS in whom BS detected supplementary skeletal injury(ies) in 34 children (Figure 1). The added value of BS after a positive RS was already reported in the literature (26–31), and its clinical usefulness may be less obvious than the added value of BS after a negative RSS. Furthermore, performing BS in children with a negative RSS and in all children with suspected physical abuse would increase the number of BS procedures required and the NNT.

### Conclusions

Despite several original studies (26–31) and one narrative review (18) concluding that RSS and BS are complementary tests, discrepancies remain between international guidelines for the indication of BS in the diagnostic work-up of suspected physical abuse in children (15, 16, 36). There is a consensus that BS should not replace RSS given its lack of sensitivity for classical metaphyseal injuries. A strategy of no BS is highly arguable given the available data on its potential for reclassifying inflicted skeletal injury and the high stakes of missing child physical abuse in relation to the risk of recidivism (estimated at 35% to 50% in the literature) (44–46) and an overall increased risk of morbi-mortality (2–5). A strategy of systematic BS after RSS, whatever the RSS results, would lead to many useless BS procedures with doubtful clinical consequences and additional invasiveness, irradiation, and costs. The add-on strategy that we evaluated for the first time in this study would benefit the reclassification potential of BS while keeping the number of children exposed to the negative effects of BS (pain, additional radiation exposure, and costs) as low as possible. Prospective multicenter studies are needed to confirm these findings.

## Data Availability Statement

The raw data supporting the conclusions of this article will be made available by the authors, without undue reservation.

## Ethics Statement

The studies involving human participants were reviewed and approved by Direction de la Recherche, Département promotion, cellule recherche non-interventionnelle of Nantes University Hospital. Written informed consent from the participants' legal guardian/next of kin was not required to participate in this study in accordance with the national legislation and the institutional requirements.

## Author's Note

Some results were presented orally on June 18, 2019 at the Second National Congress of the French Society of Pediatric Forensic Pathology in Nantes, France.

## Author Contributions

FB, EL, CG-L, and MC: conception of study. FB, EL, NV, FS, TE, CG-L, JC, and MC: design of study. FB, CP, and CG-L: data management. FB, JC, and MC: data analysis. FB and MC: drafting of the manuscript. EL, CP, NV, FS, TE, JC, and CG-L: revising of the manuscript. FB, CP, EL, NV, FS, TE, MC, and CG-L: final approval of the version to be published. All authors contributed to the article and approved the submitted version.

## Conflict of Interest

The authors declare that the research was conducted in the absence of any commercial or financial relationships that could be construed as a potential conflict of interest.
