# Supplementary material for: Bone Scintigraphy After a Negative Radiological Skeletal Survey Improves the Detection Rate of Inflicted Skeletal Injury in Children
Source: Front Pediatr. 2020 Sep 25;8:498. doi: 10.3389/fped.2020.00498 (PMC7545028; doi:10.3389/fped.2020.00498)
Supplement: Supplementary file 2 [file Table_2.docx]

**Appendix 2. Number needed to test**

Children underwent both a radiological survey (RSS) and bone scintigraphy (BS). We could build the corresponding contingency table:

|  | **BS** | |  |
| --- | --- | --- | --- |
| **RSS** | **Positive** | **Negative** | **Sum** |
| **Positive** | *a* | *b* | *a + b* |
| **Negative** | *c* | *d* | *c + d* |
| **Sum** | *a*+ *c* | *b* + *d* | *a* + *c* + *b* + *d* |

The number needed to test (NNT) with BS (i.e., number of children with a negative RSS who needed to undergo a BS to detect one additional child with skeletal injury) is defined as the inverse of the positivity rate of BS in children with a negative RSS:

$$Positivity rate(BS if RSS negative)=\frac{c}{(c+d)}$$

$$NNT=\frac{1}{c/(c+d)}=\frac{c+d}{c}$$

The boundaries of the 95%CI were inversed to obtain the 95%CI of the NNT.
