# Supplementary material for: Bone Scintigraphy After a Negative Radiological Skeletal Survey Improves the Detection Rate of Inflicted Skeletal Injury in Children
Source: Front Pediatr. 2020 Sep 25;8:498. doi: 10.3389/fped.2020.00498 (PMC7545028; doi:10.3389/fped.2020.00498)
Supplement: Supplementary file 3 [file Table_3.docx]

**Appendix 3. Characteristics of included and not included children who underwent radiological skeletal survey (RSS) with or without bone scintigraphy (BS)**

|  |  | **Underwent RSS and BS within 96 hr (N=140)** | |  | **Underwent only RSS (N=120)** | |  | **p-value^*^** |  |
| --- | --- | --- | --- | --- | --- | --- | --- | --- | --- |
|  |  | **n** | **Median (IQR**^†^**) or %** |  | **n** | **Median (IQR)  or %** |  |  |  |
| **Age at the time of the imaging investigation (months)** |  | 140 | 6 (3-8) |  | 120 | 6 (2-10) |  | 0.47 |  |
| **Sex** |  |  |  |  |  |  |  | 0.98 |  |
| Boys |  | 75 | 53.6 |  | 64 | 53.3 |  |  |  |
| Girls |  | 65 | 46.4 |  | 56 | 46.7 |  |  |  |
| **Reason for suspecting physical abuse**^‡^ |  |  |  |  |  |  |  | <0.01 |  |
| Sentinel injuries^§^ |  | 53 | 37.9 |  | 45 | 37.5 |  | 0.97 |  |
| *Bruises* |  | 49 |  |  | 39 |  |  |  |  |
| *Burns* |  | 4 |  |  | 3 |  |  |  |  |
| *Intra-oral injuries* |  | 0 |  |  | 3 |  |  |  |  |
| Index skeletal injuries |  | 61 | 43.6 |  | 37 | 30.8 |  | 0.15 |  |
| Intracranial injuries |  | 34 | 24.3 |  | 12 | 10 |  | 0.01 |  |
| Others^\|\|^ |  | 9 | 7.4 |  | 41 | 34.2 |  | <0.01 |  |
| * χ^2^ or Student *t* test  † IQR = interquartile range  ‡ Results do not total 100% because some children had several reasons for suspecting physical abuse  § Including bruises, burns and intra-oral injuries \|\| Including confession of physical abuse, physical abuse of a sibling and physical abuse suspicion of variable origins (e.g. domestic violence, drunk parents at the time of the consultation, suspicion of physical abuse by a mothers' foster home, abandonment, neglect, undernutrition, drug intoxication, fall etc.) | | | | | | | | | |
