# Supplementary material for: Bone Scintigraphy After a Negative Radiological Skeletal Survey Improves the Detection Rate of Inflicted Skeletal Injury in Children
Source: Front Pediatr. 2020 Sep 25;8:498. doi: 10.3389/fped.2020.00498 (PMC7545028; doi:10.3389/fped.2020.00498)
Supplement: Supplementary file 4 [file Table_4.docx]

**Appendix 4. Sensitivity analyses**

|  | |  | **Detection rates with the index skeletal injury included in the RSS^*^ result (N=140)** | | |  |  | **Detection rates with the index skeletal injury excluding the RSS result (N=140)** | | | |  |  | **Detection rates including children with interval between tests ≤24 hr (N=106)** | | | | |  | |
| --- | --- | --- | --- | --- | --- | --- | --- | --- | --- | --- | --- | --- | --- | --- | --- | --- | --- | --- | --- | --- |
|  | |  | **n** | **Detection rate %** | **95% CI** | **p-value**† |  | **n** | **Detection rate %** | **95% CI** | |  | **p-value** | **n** | **Detection rate %** | | **95% CI** | | **p-value** | |
| **RSS alone** | |  | 69 | 49 | (41-58) |  |  | 31 | 22 | (16-30) | |  |  | 55 | 52 | | (42-62) | |  | |
| **Add-on BS**‡ | |  | 81 | 58 | (50-66) |  |  | 50 | 36 | (28-44) | |  |  | 64 | 60 | | (50-70) | |  | |
| **Absolute increase** | |  | 12 | 9 | (4-14) | 0.001 |  | 19 | 14 | (8-20) | |  | <0.001 | 9 | 8 | | (4-16) | | <0.001 | |
| **Number needed to test** | |  | - | 6 | (4-11) |  |  | - | 6 | (4-9) | |  |  | - | 6 | | (3-12) | |  | |
|  | * RSS = radiological skeletal survey † McNemar test  ‡ BS = bone scintigraphy | | | | | | | | | |  | | | | |  |  |  | |  |
